# Complicated Brucellosis With Multisystem Involvement: Two Case Reports

**DOI:** 10.7759/cureus.15950

**Published:** 2021-06-27

**Authors:** Irfan Varikkodan, Vamanjore A Naushad, Nishan K Purayil, Joe Mathew, Farooq M Alrawi

**Affiliations:** 1 General Internal Medicine, Hamad Medical Corporation, Doha, QAT; 2 College of Medicine, Qatar University, Doha, QAT; 3 Internal Medicine, Weill Cornell, Doha, QAT; 4 Clinical Medicine, College of Medicine, Qatar University, Doha, QAT; 5 Clinical Medicine, Weill Cornell, Doha, QAT

**Keywords:** brucella, brucellosis, spinal brucellosis, epidural abscess, epididymo- orchitis

## Abstract

Brucellosis is a zoonotic infection caused by facultative intracellular bacteria of the genus Brucella. The ability of the organism to invade both phagocytic and non-phagocytic cells and survive in the intracellular environment makes brucellosis a systemic infection that can affect various organs of the body. Complications of brucellosis occur when the infection involves one or more focal body sites. Early identification of complications of brucellosis and initiation of appropriate treatment is the key to a better outcome.

Here we present two cases of complicated brucellosis, both having multiple body site involvement.

## Introduction

Brucellosis is a zoonotic infection transmitted to humans from infected animals by ingestion of food products or by contact with tissue or fluids. It is the most common bacterial zoonosis worldwide [1] . Brucellosis is also known as Mediterranean fever, Malta fever, or undulant fever. Brucellosis has been an emerging disease since the discovery of *Brucella melitensis* by Sir David Bruce in 1887. Approximately 500,000 cases are reported annually worldwide [2].

Brucellosis usually manifests as gradual onset fever, malaise, and joint pains. Brucellae are gram negative coccobacilli. Four species of Brucellae organisms are identified in causing human infection (*B. melitensis*, *B. abortus*, *B. suis*, *B. canis*), the most common being *Brucella melitensis* [1]. Osteoarticular manifestation is the most common complication of brucellosis and usually presents as arthritis, sacroiliitis, spondylitis, or osteomyelitis [3,4]. A cervical epidural abscess is a rare complication of spinal brucellosis. 

## Case presentation

Case 1

A 27-year-old gentleman from Bangladesh working in a sheep farm was admitted with fever, neck pain, and back pain with associated nausea of five days duration. He denied headache, photophobia, or vomiting. There were no motor or sensory complaints, bowel or bladder incontinence, or symptoms suggestive of cranial nerve involvement. There were no respiratory or gastrointestinal symptoms. His past medical history revealed taking incomplete treatment for brucellosis with rifampicin and doxycycline six months prior.

On admission, he was febrile (temperature: 38.4◦celsius), and other vital signs were normal. The central nervous system examination was normal except for neck stiffness. Other system examination was unremarkable. Initial laboratory investigations showed mildly elevated hepatic transaminases, alanine aminotransferase (ALT) - 57U/L (normal range: 0-41), aspartate aminotransferase (AST) - 66 U/L (normal range: 0-40) and C-reactive protein (CRP) - 49.8 mg/L (normal range: 0-5). Cerebrospinal fluid (CSF) analysis showed clear fluid, white blood cell count-8, red blood cells - 1070, protein - 0.78 g/L. CSF sample was also sent for viral study and culture (Table 1).

**Table 1:** Laboratory results. WBC: white blood cell count; RBC: red blood cell count; ALP: alkaline phosphatase; ALT: alanine aminotransferase; AST: aspartate aminotransferase; CRP: C-reactive protein; CSF: cerebrospinal fluid; IgM: immunoglobulin M; IgG: immunoglobulin G.

Blood tests	Result	Normal range
WBC	8200/µL	4000-10000
Hb	13.9 g/dL	13-17
Platelets	160 x 10^3^/uL	150000-400000
Neutrophil count	3800/µL	2000-7000
Lymphocyte count	3800/µL	1000-3000
Urea	3 mmol/L	2.8-8.1
Creatinine	59 µmol/L	62-106
Sodium	134 mmol/L	136-145
Potassium	4.5 mmol/L	3.5-5.1
Calcium	2.25 mmol/L	2.15-2.5
Total bilirubin	14 µmol/L	0-21
Total protein	86 g/L	66-87
Albumin	37 g/L	35-52
ALP	148 U/L	40-129
ALT	57 U/L	0-41
AST	66 U/L	0-40
CRP	49.8 mg/L	0-5
Brucella Ab IgM	Positive	
Brucella Ab IgG	Positive	
Brucella abortus titer	Positive 1:640	
Brucella melitensis titer	Positive 1: 1280	
Blood culture	Brucella species	
CSF analysis		
WBC	8/µL	0-5
RBC	1070/µL	0-2
Neutrophil	9%	
Lymphocyte	91%	
Glucose	2.73 mmol/L	2.22-3.89
Protein	0.78 g/L	0.15-0.45
Viral study	Negative	
Culture	No growth	

In view of mild leukocytosis and mildly elevated protein in CSF, a possibility of aseptic meningitis / partially treated bacterial meningitis was considered, and the patient was started on intravenous ceftriaxone, vancomycin, and acyclovir. Keeping in mind his profession and history of partially treated brucella infection in the past, blood was sent for culture and Brucella antibodies.

**Figure 1:**
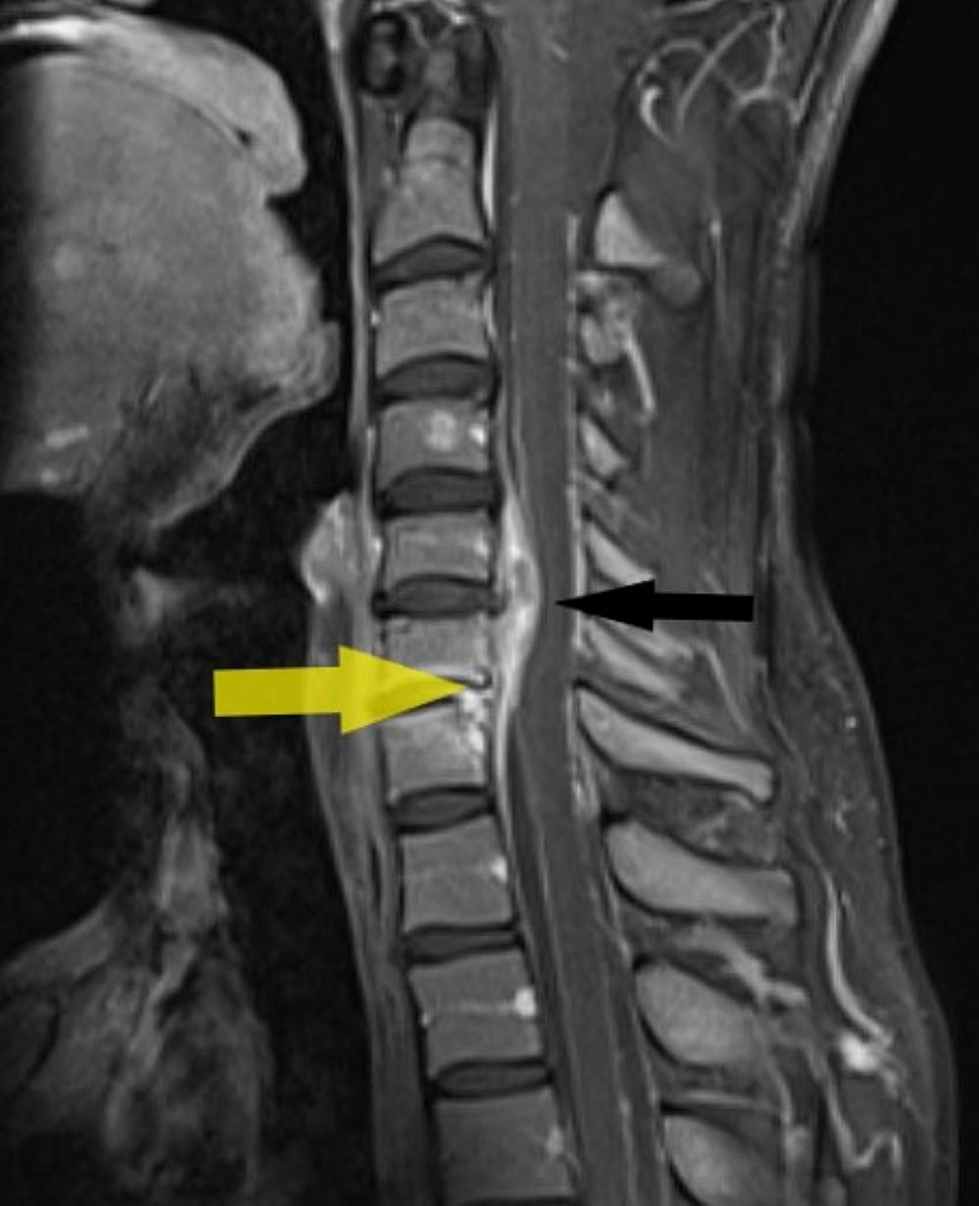
MRI showing C6/C7 infective spondylodiscitis (yellow arrow) with enhancing epidural soft tissue/phlegmon from C5 through C7 (black arrow) causing mild cervical cord compression.

Since the patient had persistent neck pain and cervical vertebral tenderness, magnetic resonance imaging (MRI) of the brain and cervical spine was performed, which revealed C6/C7 infective spondylodiscitis with enhancing epidural soft tissue/phlegmon from C5 through C7, causing mild cervical cord compression. There was no spinal cord edema or myelopathy changes noted in MRI (Figure 1).

The neurosurgical team was consulted, and they suggested conservative management and observation as there was no neurological deficit noted at that point in time. CSF analysis for viral study and tuberculosis work up by smear, polymerase chain reaction (PCR), and culture were negative. Blood culture grew Brucella species on the fifth day of hospital admission, and later, IgG and IgM brucella antibodies were reported to be elevated (*Brucella melitensis* 1:1280, *Brucella abortus* 1:640). He was initiated on doxycycline, rifampicin, and gentamicin. In view of the CSF findings, ceftriaxone was continued as there was a possibility of co-existing neurobrucellosis.

**Figure 2:**
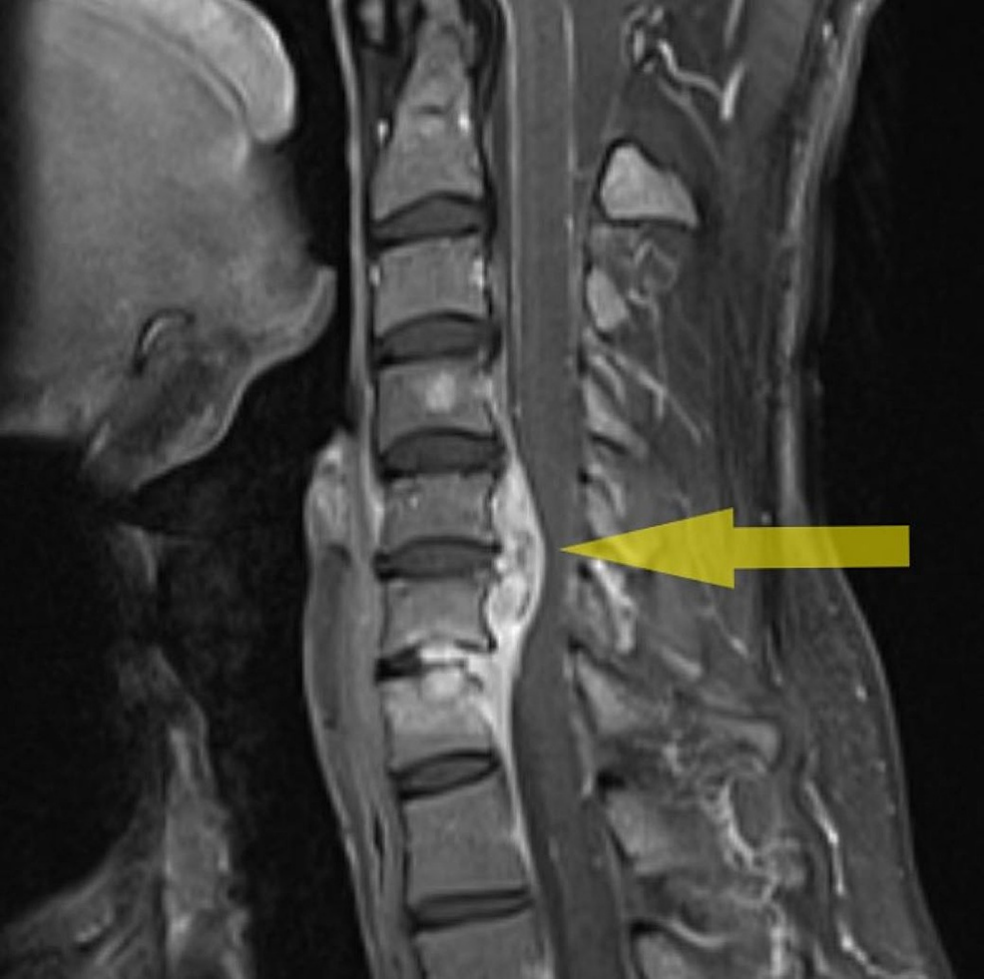
Repeat MRI of the cervical spine showing the significant progression of anterior epidural soft tissue mass with spinal cord compression (yellow arrow).

During the hospital stay, the patient developed new neurological signs in the form of exaggerated deep tendon reflexes in both lower limbs. However, there was no limb weakness or sensory deficit. Repeat MRI of the cervical spine showed significant anterior epidural soft tissue mass progression with spinal cord compression (Figure 2).

**Figure 3:**
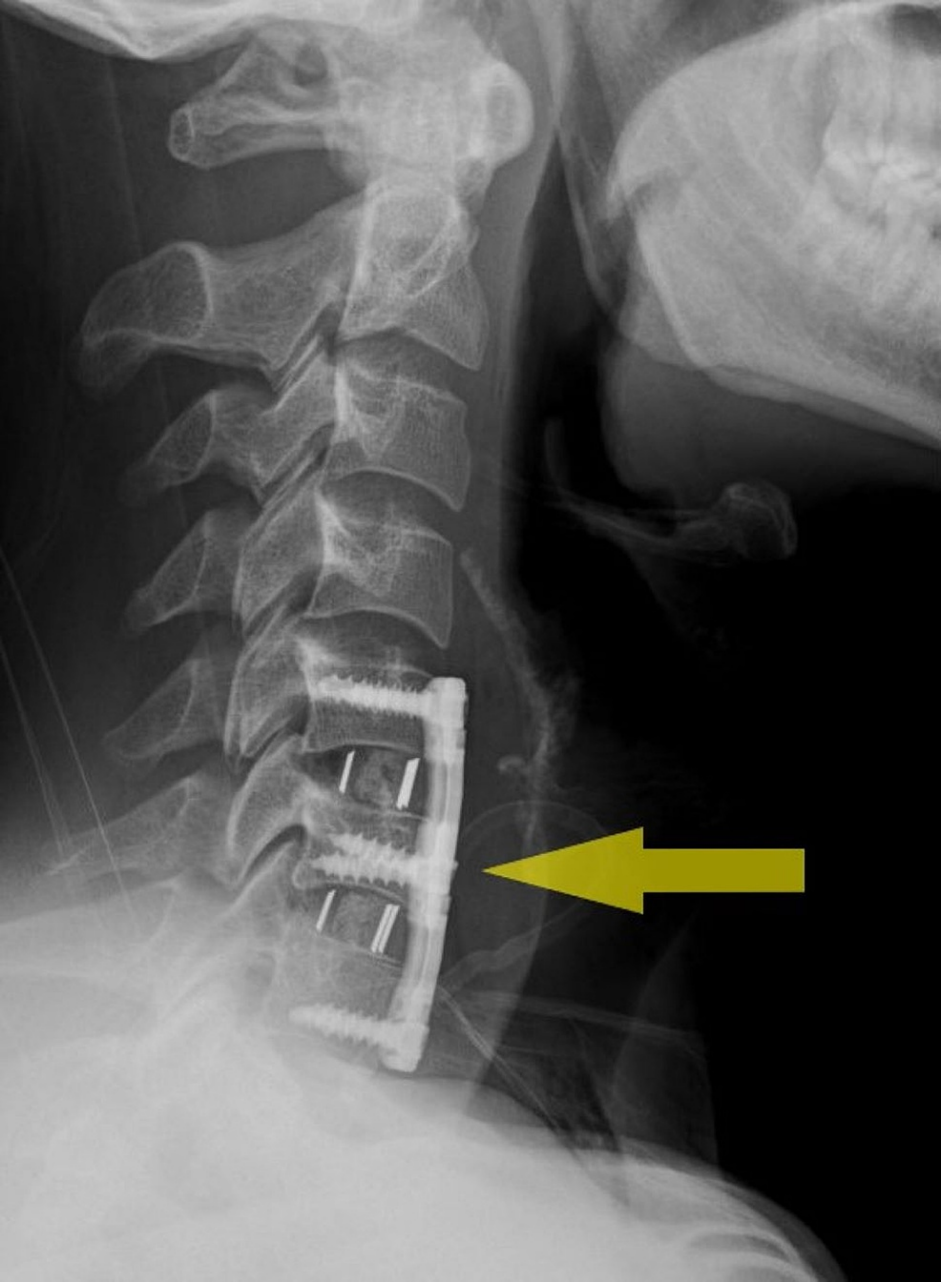
Post operative cervical spine X-ray showing vertebral fixation (yellow arrow).

In view of the progression of anterior epidural soft tissue mass, spinal cord compression, and features of myelopathy, the patient underwent C5/C6 and C6/C7 anterior cervical discectomy, evacuation, and resection of anterior epidural collection and fixation (Figure 3).

His postoperative period was uneventful. Culture from epidural collection reported no growth of bacteria or fungi. TB-PCR and AFB smear were also negative. Histopathological examination of the resected tissue showed bony tissue infiltrated with acute and chronic lymphocytic inflammation. No granuloma or malignancy was detected. Gentamicin was continued for one week and ceftriaxone for four weeks whereas oral doxycycline and rifampicin were continued for 12 weeks. The patient was discharged home after four weeks and he was asymptomatic at the time of discharge.

Case 2

A 55 -year-old Syrian gentleman presented with complaints of low back pain of six months duration which gradually increased in severity over weeks. He denied trauma at any point in time. His past history included diabetes mellitus, hypertension, coronary artery disease, and benign prostatic hypertrophy, and he was on regular medication. The pain was more on movement like walking, bending forward, and getting up from a chair; however, he could walk with pain. There was no radiation of pain, motor or sensory complaints in lower limbs, bowel or bladder complaints. He denied fever, joint pain or swelling, neck pain, respiratory or gastrointestinal symptoms. He took medical consultation a few times before for the above complaint and was prescribed analgesics but had only partial relief. A lumbar spine X-ray, which was done six months prior, was reported to be normal. He reported a weight loss of 15 kilograms over the previous four months, which he claims to be intentional.

The patient also complained of intermittent pain over the right testis over the last six months. Testicular ultrasound done six months prior reported right-sided epididymitis, and he was prescribed oral antibiotics for two weeks. However, he did not get complete relief, and the pain persisted. He denied contact with animals but gave history of drinking raw camel milk two years prior to the current admission. 

Physical examination revealed tenderness over the lower lumbar spines. There was no spinal deformity or local swelling over the back, and there was no neurological deficit. Scrotal examination showed tenderness over the right testis with swelling and tenderness over the right epididymis. The patient was afebrile during his hospital stay. Other system examination was unremarkable.

Laboratory investigations were normal except for mild microcytic hypochromic anemia (Hb 12.7 g/dl) and mildly elevated CRP of 9.1 mg/L (normal range: 0-5) (Table 2).

**Table 2:** Laboratory results. WBC: white blood cell count; Hb: hemoglobin; MCV: mean corpuscular volume; MCH: mean corpuscular hemoglobin; IgM: immunoglobulin M; IgG: immunoglobulin G.

Blood tests	Result	Normal range
WBC	6800 /µL	4000-10000
Hb	12.7 g/dL	13-17
MCV	79 fL	83-101
MCH	26.9 pg	27-32
Platelets	217 x 10^3/^uL	150000-400000
Neutrophil count	3400/µL	2000-7000
Lymphocyte count	2800/µL	1000-3000
Urea	6.3 mmol/L	2.8-8.1
Creatinine	93 µmol/L	62-106
Sodium	138 mmol/L	136-145
Potassium	4 mmol/L	3.5-5.1
Calcium	2.31 mmol/L	2.15-2.5
Total bilirubin	8 µmol/L	0-21
Total protein	78 g/L	66-87
Albumin	39 g/L	35-52
Alkaline phosphatase	86 U/L	40-129
Alanine aminotransferase	28 U/L	0-41
Aspartate aminotransferase	37 U/L	0-40
C-reactive protein	9.1 mg/L	0-5
Brucella Ab IgM	Negative	
Brucella Ab IgG	Positive	
Blood culture	Brucella melitensis	

**Figure 4:**
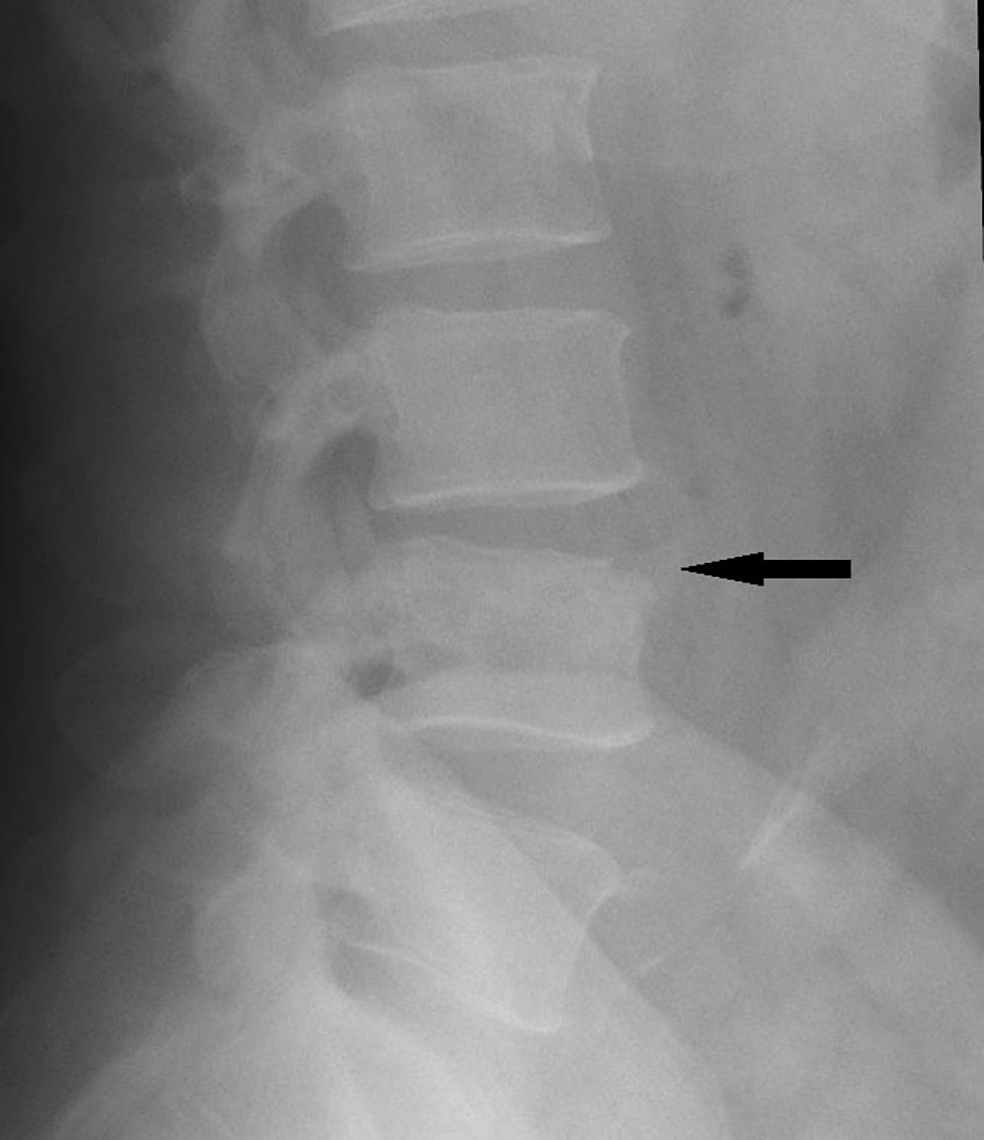
Lumbar spine X-ray showing small lytic foci involving the superior half of the L4 vertebral body with an ill-defined cortex along the anterosuperior aspect (black arrow), and mild reduction of L3-4-disc space.

Lumbar spine X-ray was repeated, which showed small lytic foci involving the superior half of the L4 vertebral body with an ill-defined cortex along anterosuperior aspect and mild reduction of L3-L4 disc space (Figure 4 ).

**Figure 5:**
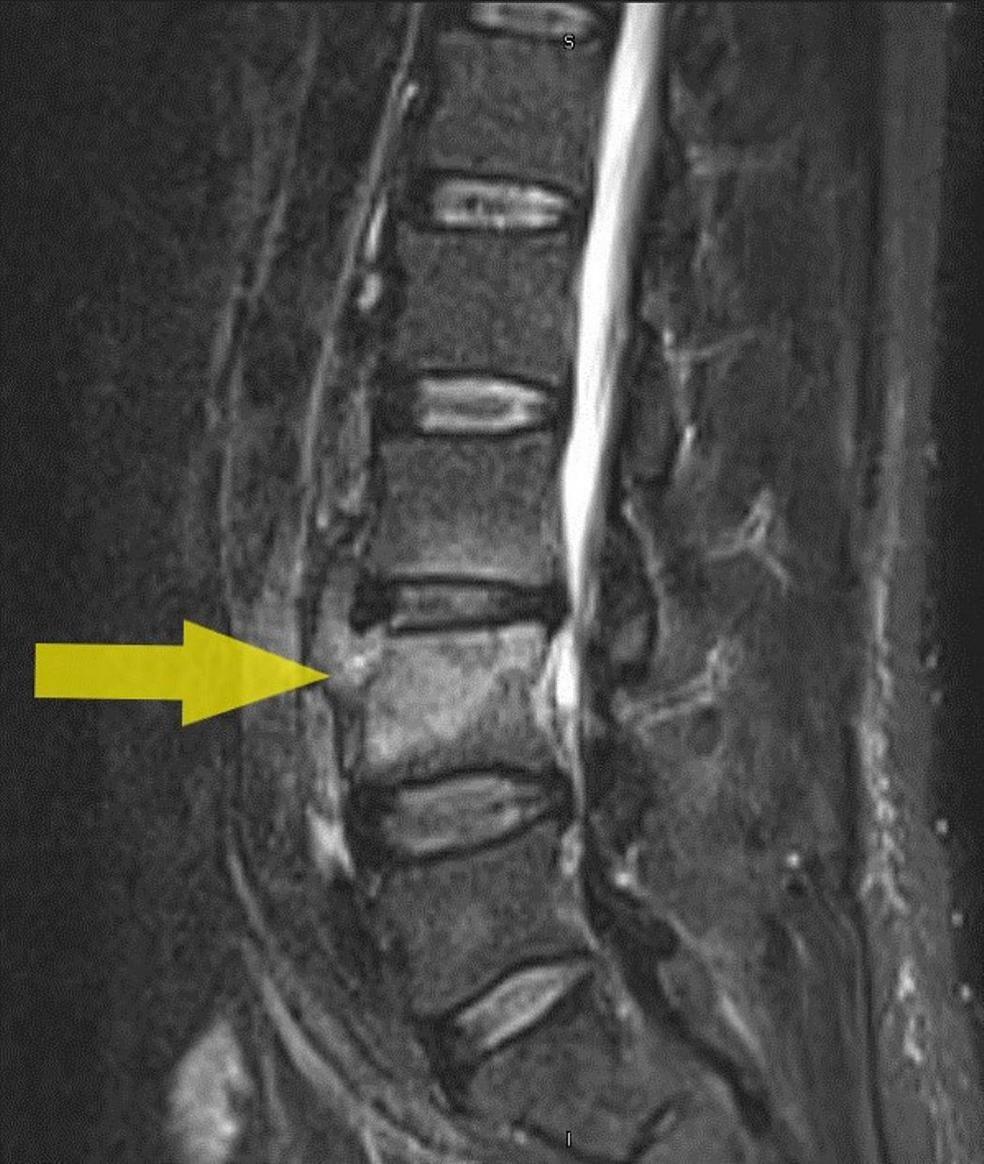
MRI Lumbar and Sacral spine showing L3/L4 prevertebral soft tissue edema/fluid and focal erosion over the anterior margin of L4 superior endplate (yellow arrow).

MRI spine showed L3/4 prevertebral soft tissue edema/fluid with focal erosion along the left anterior superior margin of L4 superior endplate. Spondylo degenerative changes of the lumbar spine with multiple disc lesions were also found (Figure 5).

Testicular ultrasound showed right-sided epididymo-orchitis with funiculitis. Abdominal ultrasound revealed hepatosplenomegaly (spleen 16.9 cm, liver 19.3 cm).

Based on the chronic nature of the symptoms and multisystem involvement, an infective cause of tuberculosis or brucellosis was considered. The quantiferon test for tuberculosis was negative. Blood culture grew *Brucella melitensis*. Brucella IgG antibody assay was positive. A probable diagnosis of brucellosis with spondylitis and right-sided epididymo-orchitis was made. Aspiration from the spinal lesion was deferred as there was no significant fluid collection.

The patient was treated with streptomycin for two weeks and oral rifampicin and doxycycline for 12 weeks. After two weeks of treatment in the hospital, he showed good clinical improvement, with the severity of back pain decreasing significantly and complete resolution of testicular pain. Repeat blood culture was sterile. He was discharged home after two weeks, and on follow-up in the outpatient clinic after six weeks, he was doing fine.

## Discussion

Complications of brucellosis occur when the infection involves one or more focal body sites. The most common form of focal brucellosis is an osteoarticular disease, followed by genitourinary disease. It can also affect the nervous system (neurobrucellosis), cardiovascular system, gastrointestinal, respiratory, hepatobiliary system, skin, and eyes. A study done by Bosilkovski et al. on 550 patients with brucellosis reported focal brucellosis in 66 % and osteoarticular disease in 54% of patients [5] whereas, another study conducted by Colmenero et al. in 530 patients with brucellosis showed 31.9 % of patients having focal brucellosis or complications and osteoarticular disease being the most common one [6].

Osteoarticular diseases include sacroiliitis, spondylitis, and peripheral arthritis. Spondylitis is a severe complication of brucellosis. Lumbar vertebrae are most commonly involved, followed by thoracic vertebrae. Cervical vertebrae are rarely involved in spinal brucellosis. Multiple vertebral body involvement was reported to be around 9%-30 % [7,8]. Bacteremia was found in around 41%-56 % of cases of spinal brucellosis [7].

Extension of infection from spondylodiscitis can rarely result in paravertebral, prevertebral, epidural, psoas muscle abscess, and radiculitis. A past study conducted by Ulu-Kilic et al. reported that spondylodiscitis was complicated with paravertebral abscess in 13%, prevertebral abscess in 4.4%, epidural abscess in 10.2%, psoas abscess in 3.4%, and radiculitis in 2.7% of their patients with brucellosis [9].

Cervical epidural abscess is a rare complication of spinal brucellosis [10-12]. Patients with cervical and thoracic spondylitis tend to have proportionally more paravertebral/epidural masses than lumbar spondylitis [13]. Signs of brucellar epidural abscess are nonspecific but can vary from fever, spinal tenderness, motor/sensory deficit, or sphincter loss due to spinal cord compression [14]. MRI is the best modality to detect epidural abscess. It has higher sensitivity and specificity and provides more comprehensive anatomic details, especially in the soft tissue compartment. The earliest reaction in the vertebral body is bone marrow edema, characterized by low signal intensity on T1 weighted images and mainly high signal intensity on T2 weighted images. The early form of brucella spondylitis is characterized by the lysis of the anterior aspect of the superior endplate at the disco vertebral junction. MRI features of brucella spondylitis include lesions primarily involving the lumbar region, mild intervertebral disk destruction, lesions on the ventral sides of the vertebral bodies, facet joint involvement, and vertebral osteophyte formation, with no or very mild vertebral body deformation and abnormal paraspinal soft tissue signal [15].

The administration of effective antibiotics for an adequate duration is the cornerstone in treating all types of brucellosis. Uncomplicated acute brucellosis usually responds well to appropriate antibiotic therapy. The preferred treatment for uncomplicated brucellosis is oral doxycycline (100 mg orally twice daily) for six weeks combined with intramuscular streptomycin (1 gm) for two to three weeks [16]. Intravenous or intramuscular gentamicin (5 mg/kg/day) can also be used for 7-10 days instead of streptomycin. The principal alternative therapy is oral doxycycline and rifampicin (600-900 mg oral daily) for six weeks [16].

Complicated brucellosis with spondylitis (spinal brucellosis), endocarditis, and neurobrucellosis requires an extended duration of treatment. The optimal antibiotic regimen and duration of treatment for spinal brucellosis are still controversial in the literature. In general, combination therapy of doxycycline for 12 weeks plus streptomycin for two to three weeks is preferred [17-19]. Three drug regimens of doxycycline and rifampicin for 12 weeks combined with streptomycin (two to three weeks) or gentamicin (7-10 days) have also been used. An observational study showed a higher frequency of clearance of Brucella DNA from blood with triple therapy [19]. However, side effects, possible drug-drug interaction, patient’s clinical and radiological response should be addressed when choosing the antibiotics and their duration [9]. Surgical treatment is indicated in case of neurological deficit, spinal instability, and non-responsiveness to antibiotic therapy [20].

The first patient in our report had spinal Brucellosis with cervical epidural abscess. In view of the CSF findings of mild leukocytosis and elevated protein, associated meningitis (neurobrucellosis) was also considered.

Genitourinary involvement can occur in up to 2%-20% of patients with brucellosis [21]. Epididymo-orchitis is the most common presentation. It can also cause prostatitis and, less commonly, testicular abscess. Cystitis, interstitial nephritis, glomerulonephritis, renal abscess, and tubo ovarian abscess are other rare manifestations. Epididymo-orchitis usually present as scrotal pain and swelling with or without fever. It is more commonly unilateral. They can also have associated urinary symptoms, but urine analysis and culture will be normal in most patients. The medical treatment for epididymo-orchitis is similar to that of uncomplicated brucellosis. Orchidectomy may be rarely required in a testicular abscess or orchitis unresponsive to medical therapy [21,22].

The second patient in our case report had lumbar spondylitis and epididymo-orchitis as a complication of brucellosis. In view of spondylitis, he was treated with an extended regimen of doxycycline and rifampicin for 12 weeks duration and streptomycin for two weeks duration, and the patients' condition improved with the treatment.

## Conclusions

Although Brucellosis can affect various organ systems of the body, simultaneous involvement of more than one organ is uncommon. Brucellosis should also be considered as an etiology of spinal epidural abscess, especially in a patient from endemic regions or if the patient had contact with animals or animal products. Early diagnosis and initiation of appropriate antibiotics for the optimum duration is the mainstay in treating brucellosis and preventing complications. Early identification of complications and prompt intervention is the key to a better outcome.
